# Transformer and group parallel axial attention co-encoder for medical image segmentation

**DOI:** 10.1038/s41598-022-20440-z

**Published:** 2022-09-27

**Authors:** Chaoqun Li, Liejun Wang, Yongming Li

**Affiliations:** College of Information Science and Engineering, Xinjiang University, Ürümqi, 830046 China

**Keywords:** Medical research, Engineering

## Abstract

U-Net has become baseline standard in the medical image segmentation tasks, but it has limitations in explicitly modeling long-term dependencies. Transformer has the ability to capture long-term relevance through its internal self-attention. However, Transformer is committed to modeling the correlation of all elements, but its awareness of local foreground information is not significant. Since medical images are often presented as regional blocks, local information is equally important. In this paper, we propose the GPA-TUNet by considering local and global information synthetically. Specifically, we propose a new attention mechanism to highlight local foreground information, called group parallel axial attention (GPA). Furthermore, we effectively combine GPA with Transformer in encoder part of model. It can not only highlight the foreground information of samples, but also reduce the negative influence of background information on the segmentation results. Meanwhile, we introduced the sMLP block to improve the global modeling capability of network. Sparse connectivity and weight sharing are well achieved by applying it. Extensive experiments on public datasets confirm the excellent performance of our proposed GPA-TUNet. In particular, on Synapse and ACDC datasets, mean DSC(%) reached 80.37% and 90.37% respectively, mean HD95(mm) reached 20.55 and 1.23 respectively.

## Introduction

As China's population ages, people's awareness of diseases has deepened, and health consciousness has constantly improved. The diagnosis of diseases requires doctors to analyze and discriminate CT or MR maps, etc., which is bound to generate a mass of work. Therefore, the use of computers to assist physicians in diagnosis has become a matter of urgency. Computer-aided diagnosis technology has comprehensive applications and research values for medical studies, pathology analysis, and image information processing.

Medical image segmentation plays an extremely important role in disease diagnosis and clinical medicine. Early medical image segmentation systems were mainly built based on traditional image segmentation algorithms. Such as edge detection-based methods and region-based methods. Later, with the rapid development of computer technology, deep learning algorithms based on Convolutional Neural Network (CNN)^1^ have made breakthroughs. UNet^2^ is a medical image segmentation network based on CNN. It consists of encoder-decoder and has been proven effective for many different segmentation tasks. Examples include cardiac segmentation by magnetic resonance (MR)^3^, organ segmentation by computed tomography (CT)^4–6^, and polyp segmentation by colonoscopy video^7^. Despite the dominance of U-Net in medical image segmentation, it and its variants^7–9^ face the same problems that CNN-like models (including fully convolutional nets (FCNs)^10^) cannot avoid: lack of long-term global correlation modeling capabilities. The main reason is that CNNs extract local information simply, but they cannot measure global relevance efficiently.

Many recent works have attempted to address this problem by using Transformer encoders^11–13^. Transformer designed for sequence-to-sequence prediction^14^ originally, it’s a model based on self-attention (SA). SA is a core part of Transformer. Due to SA's ability to model the correlation between all input tokens, Transformer is able to handle global long-term dependencies. In this case, some recent works have achieved satisfactory results^13,15–19^, pure Transformer models^20^ have also emerged. Since the foreground information of medical images is usually presented as regional blocks, the local detail information is equally important to segmentation results. However, Transformer focuses on the extraction of global information but weakens local information, so it also has some disadvantages in medical image segmentation tasks. How to properly highlight foreground information, weaken background information, and how to better jointly model local information and global correlation dependence becomes the focus of our study.

In order to solve these problems, we design a new attention mechanism GPA and cite the Sparse-MLP (sMLP) proposed by Chuanxin Tang et al.^21^. We combine GPA with Transformer as encoder. GPA attention enhances the model's perception of sample axial information and weakens the background information, thus strengthens the local information of sample. At the same time, Transformer's long-range correlation modeling capability is preserved to capture global information of the sample. We further introduce sMLP to intensify the global information. The sMLP has the advantage of sparse connectivity and weight sharing with slight parameters. Through Transformer and GPA co-encoder, sMLP enhances global information modeling, we obtain more significant feature encoding capabilities for medical image segmentation.

Concretely, our contributions can be summarized as:

1. We design a new attention mechanism method: GPA. It focuses on model local information dependence. We utilize GPA and Transformer as the co-encoder.
2. We cite sMLP, which completes sparse connectivity and weight sharing. Global modeling capabilities of the network are enhanced by applying it.
3. In summary, we propose GPA-TUNet. We demonstrate its effectiveness on two different public datasets (Synapse multi-organ segmentation dataset and Automated cardiac diagnosis challenge dataset). The experimental results show that our method has many advantages over other competing methods.

The remaining content of this paper is as follows. "Related Work" Section is related work. "GPA-TUNet" Section introduces the architecture of GPA-TUNet and related modules in detail. "Experiments and Analysis" Section is experimental results and analysis. "Conclusions" section gives conclusions.

## Related work

### CNN-based methods

Edge detection and traditional machine-learning-based algorithms were the methods employed in early medical image segmentation. With the development of deep learning, UNet^2^ was proposed for medical image segmentation, which based on encoder-decoder structure. UNet has simple structure and reliable performance, so many UNet-like networks have emerged. For example, Res-Unet^22^ with residual structure, UNet +  + ^7^ with nested U-shape structure, HDC-Net[23]with hierarchical dilation convolutional. It has also been applied in 3D medical image segmentation, such as V-Net^24^. Currently, CNN-based methods have achieved great success in medical image segmentation field.

### Transformers

Transformer^14^ started with Natural Language Processing and Text Embedding^25^. Transformer has been applied not only to target detection^12^, semantic segmentation^26,27^ and image classification^11^, but also to medical image segmentation^9,28,29^. Built on the very successful Vision Transformer (ViT)^11^, TransUNet^13^ is the first Transformer-based medical image segmentation framework. It effectively combines CNN with Transformer, which is implemented for local–global correlation modeling.

### Combining CNNs with self-attention mechanisms

Many researchers have attempted to integrate self-attention into CNNs based on global modeling of all pixels of the feature mapping. For example, Wang et al.^30^ designed a nonlocal operator that is inserted into multiple internal intermediate convolutional layers. Schlemper et al.^9^ proposed additional attention gate modules integrated into skip connections based on the encoder-decoder U-shaped structure.

### Combining attention mechanisms with transformers

Attention mechanisms have been widely used in various research areas since their emergence and have resulted in many novel types of attention mechanisms^31–35^. Various attention mechanism algorithms are similar in that they all aim to highlight local foreground information while weakening background. While attention mechanisms perform well in modeling local relevance, they lack ability to model global information correlation. However, Transformer excels in measuring the relevance of all elements. Therefore, we designed a new attention mechanism and combined it with Transformer. By this means, our model not only highlights the local foreground information, but also preserves the edge information we are interested in. It has been experimentally demonstrated that GPA-TUNet well implements joint modeling of local information dependence and global relevance dependence.

There are too many methods based on attention^36,37^, self-attention^38^ and Transformer^13,39^. ^36,37^ extract features by introducing spatial attention or combination of spatial attention and channel attention. ^38^ utilizes self-attention to improve the discriminativeness of feature representations. ^13,39^ extract features by Transformer singularly. These methods have played a certain role in respective tasks, but the above methods extract features only by attention or Transformer singularly, which easily leads to the loss of global information or local foreground information of samples. Different from the above methods: (1) We open a new attention research direction starting from the axial correlation of samples and propose GPA attention, which different from channel attention or spatial attention. (2) We combine GPA and Transformer as co-encoder to reduce information loss and achieve joint modeling of local foreground information and global correlation dependencies. (3) GPA-TUNet not only highlights the local foreground information (which is very important for medical segmentation tasks), but also preserves the edge information we are interested in.

Different from the above, we effectively combine the attention mechanism with Transformer in our method to more mightily jointly model local information dependence and global correlation dependence.

## GPA-TUNet

In this section, the detail of GPA-TUNet method is described, including research motivation, the architecture of network and related modules.

### Research motivation

Recently, there are some defects in medical image segmentation method that need to be dealt with: (1) CNNs extract local information simply, but they cannot measure global relevance efficiently. (2) Transformer performs well in modeling global information but cannot extract local details well. In order to solve these problems, some scholars consider combining CNN with Transformer as hybrid encoder. Since medical images are often presented as regional blocks, foreground information is quite important for segmentation results. But these methods do not highlight the importance of foreground information in medical images. Based on the above considerations, the goal of this paper is to design an attention mechanism to highlight the importance of sample foreground information. We combine it with Transformer as co-encoder and enhance global modeling with MLP block. Therefore, local foreground information dependency and global correlation dependency can be jointly modeled for better medical image segmentation performance.

Given an image *X* ∈ $R^{C \times H \times W}$, C is the number of channels, and *H* × *W* is the spatial resolution. Our goal is to predict pixel-level segmentation maps of the same size *H* × *W*. For medical image segmentation tasks, many researchers have applied Transformer or attention mechanism to encoder singly. Unlike existing methods, we use Transformer combined with GPA attention mechanism as co-encoder. Next in this paper, we will first introduce our general framework. Then we introduce the encoder and decoder of GPA-TUNet in turn. Among them we focus on the architectural design of GPA and the GPA combines Transformer as co-Encoder approach. Necessarily, sMLP Block will be discussed.

### Overall GPA-TUNet

**Figure 1:**
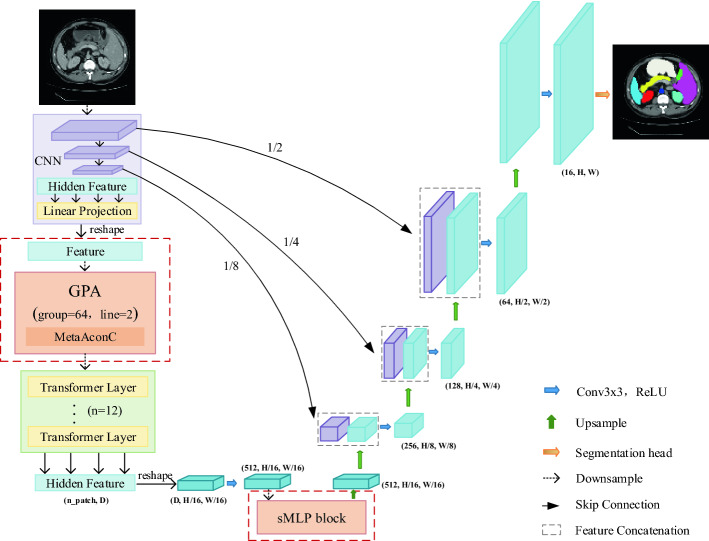
Overview of the framework. (Created by ‘Microsoft Office Visio 2013’ url: https://www.microsoft.com/zh-cn/microsoft-365/previous-versions/microsoft-vision-2013).

The overall framework of the network is shown in Fig. 1. The network is based on U-shape architecture. The encoder consists of CNN, GPA and Transformer. The decoder uses dilated convolutions and skip connections to preserve the underlying features. As shown in Fig. 1, CNN is first used to extract coarse features. Next, we use GPA to extract the local foreground information in axial direction and weaken background information, which is embedded between CNN and Transformer. Then, we transfer the feature map into Transformer to extract global correlation of samples. To further preserve global information, we introduce a module at the end of encoder (before upsampling): sMLP block. It serves to achieve sparse connectivity and weight sharing between rows and columns. The decoder is then aligned with TransUNet ^13^.

### CNN layer

For the CNN layer of our network, we adopt the same setting as TransUNet ^13^, that is, ResNet50 is used to extract rough features of samples.

### GPA layer

**Figure 2:**
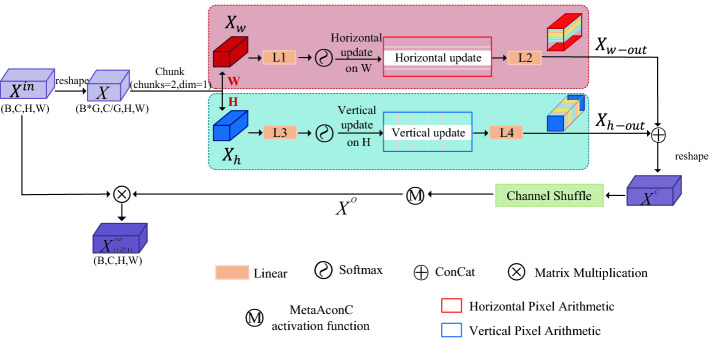
Overview of the proposed GPA Attention. (Created by ‘Microsoft Office Visio 2013’ url: https://www.microsoft.com/zh-cn/microsoft-365/previous-versions/microsoft-vision-2013).

Overview of the proposed GPA is shown in Fig. 2. As is presented, the GPA is divided equally into two branches according to the number of channels of input *X*. The upper branch implements pixel-based horizontal (width) attention and the lower branch implements pixel-based vertical (height) attention. The outputs of the two branches are fused and reshaped to obtain $X^{c}$. $X^{c}$ goes through the channel shuffle and activation function Meta-ACON^40^ module to obtain the new output $X^{o}$. The final output $X^{out}_{{\left( {GPA} \right)}}$ is obtained by multiplying $X^{o}$ with the original input $X^{in}$.

Firstly, we let $X^{in}$ ∈ $R^{B \times C \times H \times W}$ denote the collection of input tokens. After reshaping, the feature vector is $X \in R^{{\left( {B*G} \right) \times \left( {C/G} \right) \times H \times W}}$, *G* is the multiple of the channel reduction, set to 64. Then the input $X$ is divided into two branches by the number of channels to obtain $X_{w}$ and $X_{h}$ respectively, $X_{w} \in R^{{\left( {B*G} \right) \times \left( {C/2G} \right) \times H \times W}}$, $X_{h} \in R^{{\left( {B*G} \right) \times \left( {C/2G} \right) \times H \times W}}$.

The axial attention calculation is performed next. The upper branch is based on the pixel-based horizontal attention (sample width attention) calculation. The Eq. (1) is as follows:

1 $$ X_{w - out} = L_{2} \left\{ {HPA\left[ {softmax\left( {(L_{1} \left( {X_{w} } \right)} \right)} \right]} \right\} $$

where $$L_{1}$$ and $$L_{2}$$ represent the fully connected layer. $$L_{1}$$ makes the width of the image larger and keeps the height. That is, the size of $$X_{w}$$ changes from $$H \times W$$ to $$H \times W_{l}$$ after passing through $$L_{1}$$(In this paper, $$H$$ = $$ W$$ = 14, $${ }W_{l}$$ = 32). $$L_{2}$$ then reduces the size from $$H \times W_{l}$$ to $$H \times W$$. The softmax operation is set to dim = 1.

**Figure 3:**
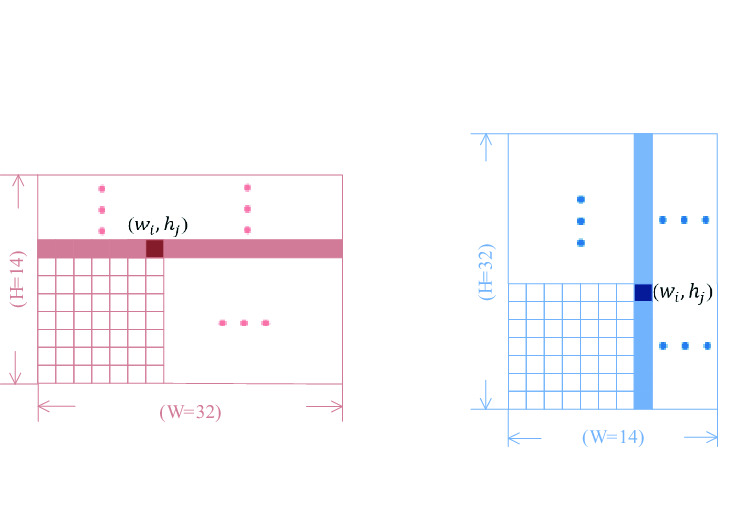
Horizontal Pixel Arithmetic and Vertical Pixel Arithmetic. (Created by ‘Microsoft Office Visio 2013’ url: https://www.microsoft.com/zh-cn/microsoft-365/previous-versions/microsoft-vision-2013).

*HPA* (Horizontal Pixel Arithmetic) indicates that the feature encoding is updated by the pixel value on the width of images. The update method is briefly illustrated through Fig. 3a. As shown in Fig. 3, Divide a picture of size $H \times W$ into several disjoint rectangular patches, each patch represents a pixel point, corresponding to a pixel value. *HPA* is to divide the pixel value of each patch by the sum of all pixel values on its row. It generates some new weights, which are assigned to each patch respectively. The horizontal attention weight update calculation Eq. (2) of the $\left( {w_{i} ,h_{j} } \right)$ th pixel on each channel is as follows:

2 $$ HPA\left( {w_{i} ,h_{j} } \right) = \mathop \sum \limits_{C = 0}^{c} \frac{{value\left( {w_{i} ,h_{j} } \right)}}{{value\left[ {\left( {w_{0} + w_{1} + \cdots w_{i} + \cdots + w_{w + 1} } \right),h_{j} } \right]}} $$

where *C* denotes the number of channels, and $value\left( {w_{i} ,h_{j} } \right)$ denotes the pixel value of point $\left( {w_{i} ,h_{j} } \right)$.

Similarly, the lower branch is a pixel-based vertical attention (sample height attention) calculation given by the following Eq. (3) and (4).

3 $$ X_{h - out} = L_{4} \left\{ {VPA\left[ {softmax\left( {(L_{3} \left( {X_{h} } \right)} \right)} \right]} \right\} $$

4 $$ VPA\left( {w_{i} ,h_{j} } \right) = \mathop \sum \limits_{C = 0}^{c} \frac{{value\left( {w_{i} ,h_{j} } \right)}}{{value\left[ {w_{i} ,\left( {h_{0} + h_{1} + \cdots h_{j} + \cdots + h_{h + 1} } \right)} \right]}} $$

where $$L_{3}$$ and $$L_{4}$$ represent the fully connected layer. $$L_{3}$$ makes the height of the image larger and keeps the width. That is, the size of $$X_{h}$$ changes from $$H \times W$$ to $$H_{l} \times W$$ after passing through $$L_{3}$$(In this paper, $$H$$ = $$ W$$ = 14,$${ }H_{l}$$ = 32). $$L_{4}$$ then reduces the size from $$H_{l} \times W$$ to $$H \times W$$. The softmax operation is set to dim = 1. As shown in Fig. 3b, *VPA* (Vertical Pixel Arithmetic) is to divide the pixel value of each patch by the sum of all pixel values on its column. The new weights are assigned to each patch respectively.

**Figure 4:**
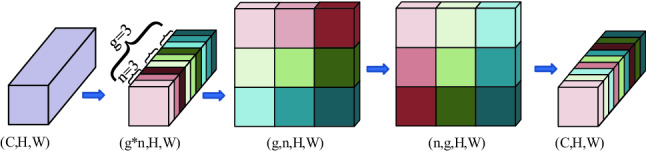
Schematic diagram of channel shuffle. (Created by ‘Microsoft Office Visio 2013’ url: https://www.microsoft.com/zh-cn/microsoft-365/previous-versions/microsoft-vision-2013).

We splice $X_{w - out} \in R^{{\left( {B*G} \right) \times \left( {C/2G} \right) \times H \times W}}$ and $X_{h - out} \in R^{{\left( {B*G} \right) \times \left( {C/2G} \right) \times H \times W}}$ in cascade and then reshape it into the original input dimension size $X^{c} \in R^{B \times C \times H \times W}$. The Eq. (5) is as follows:

5 $$ X^{c} = Re\left[ {concat\left( {X_{w - out} ,X_{h - out} } \right)} \right] $$

where $concat$ and $Re$ represent concat by number of channels and reshape feature vector respectively. In order to achieve information flow between features of different groupings, we perform the channel shuffle operation. The channel shuffle schematic is shown in Fig. 4. First assume that the number of input channels *C* is divided into g groups, and each group contains n channels. Split the channels *C* into (g, n) two dimensions. Then transpose (g, n) into (n, g). Finally, reshape the (n, g) dimensions to one dimension *C* (*C* = n*g). In this way, information can flow between different groups.

For the activation of feature vectors, we adopt a novel activation function, *ACON-C*^40^. In neural networks, many common activation functions are in the form of max ($\eta_{a} \left( x \right)$*,* $\eta_{b} \left( x \right)$) function (e.g., ReLU max(x,0) and its variants) where $\eta_{a} \left( x \right)$ and $\eta_{b} \left( x \right)$ denote linear functions. The Eq. (6) proposed by Ningning Ma et al.^40^ to approximate the activation function.

6 $$ S_{\beta } \left( {\eta_{a} \left( x \right), \eta_{b} \left( x \right)} \right) = \left( {\eta_{a} \left( x \right) - \eta_{b} \left( x \right)} \right) \cdot \sigma \left[ {\beta \left( {\eta_{a} \left( x \right) - \eta_{b} \left( x \right)} \right)} \right] + \eta_{b} \left( x \right) $$

where *σ* is the sigmoid function, *β* is the switching factor. The authors used a dual-parameter function to further propose the *ACON-C* activation function. As follow Eq. (7):

7 $$ f_{ACON - C} \left( x \right) = S_{\beta } \left( {p_{1} x,p_{2} x} \right) = \left( {p_{1} - p_{2} } \right)x \cdot \sigma \left[ {\beta \left( {p_{1} - p_{2} } \right)x} \right] + p_{2} x $$

Formally, $$\eta_{a} \left( x \right) = p_{1} x$$, $$\eta_{b} \left( x \right) = p_{2} x$$($$p_{1} \ne p_{2}$$).

*ACON-C*^40^ enable to adaptively choose whether to activate neurons. It controls whether the neuron is activated by the value of *β* (*β* is 0, i.e., not activated). The design space of the adaptive function of β utilizes channel-wise, i.e., channel space. *H*, *W* dimensions are first averaged separately and then passed through two convolutional layers so that all pixels in each channel share a weight. The Eq. (8) is as follows:

8 $$ \beta = \sigma W_{1} W_{2} \mathop \sum \limits_{h = 1}^{H} \mathop \sum \limits_{w = 1}^{W} x_{c,h,w} $$

where *σ* is the sigmoid function, and $W_{1}$ and $W_{2}$ are two convolution operations respectively. $W_{1}$ ∈ $R^{{C \times \left( {C/r} \right)}}$, *C* is the dimension of the input and *C/r* is the dimension of the output. $W_{2}$ ∈ $R^{{\left( {C/r} \right) \times C}}$, The *C/r* is the dimension of the input and *C* is the dimension of the output. To save the number of parameters, a scaling parameter *r* is added between $W_{1} \left( {C,C/r} \right)$ and $W_{2} \left( {C/r,C} \right)$ and set to 16.

We denote the feature vector after activation as $X^{o} \in R^{B \times C \times H \times W}$. As shown in Eq. (9):

9 $$ X^{o} = ACON\left[ {shuffle\left( {X^{c} } \right)} \right] $$

Matrix multiplication of $X^{o}$ with the original input $X^{in} \in R^{B \times C \times H \times W}$ to get the final output feature vector, which is the final output of GPA. As shown in Eq. (10).

10 $$ X^{out}_{{\left( {GPA} \right)}} = X^{in} X^{o} $$

### Transformer layer

**Figure 5:**
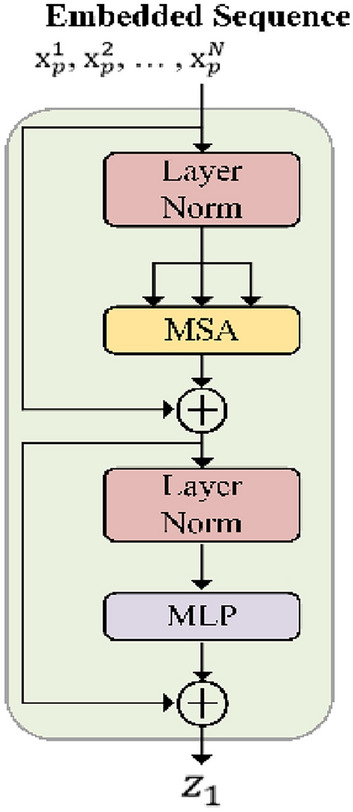
Schematic of the Transformer layer. (Created by ‘Microsoft Office Visio 2013’ url: https://www.microsoft.com/zh-cn/microsoft-365/previous-versions/microsoft-vision-2013).

We further hand over the GPA output to the Transformer for processing. The Transformer sets to 12 layers, and the structure of each Transformer layer is shown in Fig. 5.

The feature vectors are first patch embedding in Transformer layer. We map the vectorized patches $x_{p}$ to the potential *D*-dimensional embedding space by virtue of a linear projection which is trainable. We preserve location information by adding location embeddings to patches, thereby encoding spatial information. As follows Eq. (11):

11 $$ z_{0} = \left[ {{\text{x}}_{p}^{1} E;{\text{ x}}_{p}^{2} E;{ ,} \ldots {,};{\text{ x}}_{p}^{N} E} \right] + { }E_{pos} $$

where ${\text{E}} \in R^{{\left( {P^{2} \cdot C} \right) \times D}}$ is the patch embedding projection, and $E_{pos} \in R^{N \times D}$ denotes the position embedding.

The Multi-head Self Attention (*MSA*) and Multi-Layer Perceptron (*MLP*) blocks of *l* layers together form the Transformer encoder (Eq. (12) and (13)). Output of the $l$-th layer can be written as follows:

12 $$ z_{l}^{\prime } = MSA\left( {LN\left( {z_{l - 1} } \right)} \right) + z_{l - 1} $$

13 $$ z_{l} = MLP\left( {LN\left( {z_{l}^{\prime } } \right)} \right) + z_{l}^{\prime } $$

where $$LN\left( \cdot \right)$$ denotes the layer normalization operator and $$z_{l} $$ is the encoded image representation.

The joint encoder leverages GPA to highlight local foreground information and diminish background information, while retaining Transformer's powerful ability to measure the relevance of global elements. That is, our GPA-TUNet adequately and rationally models local correlation and global correlation. Therefore, our encoded feature maps not only reinforce the salient features in different directions of samples, but also preserve the edge information we are interested in.

### sMLP block

**Figure 6:**
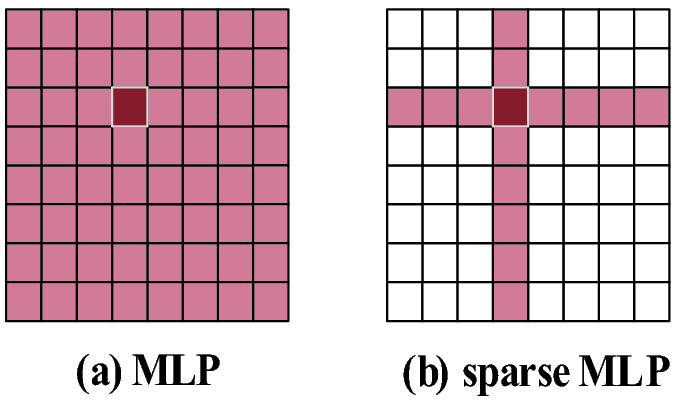
sMLP reduces the computational complexity of MLP. (Created by ‘Microsoft Office Visio 2013’ url: https://www.microsoft.com/zh-cn/microsoft-365/previous-versions/microsoft-vision-2013).

Chuanxin Tang et al. proposed Sparse MLP (sMLP)^21^ based on the MLP-based vision model, replacing the MLP module in the token-mixing step with a new sMLP module. For a 2D image, sMLP applies 1D MLP along the image height and width, so the parameters are shared between rows or columns. As shown in Fig. 6a, the dark-colored token interacts with all other light-colored tokens in a single MLP layer. However, in an sMLP layer (as shown in Fig. 6b), the dark-colored token only interacts with horizontal and vertical light-colored tokens it is on.

**Figure 7:**
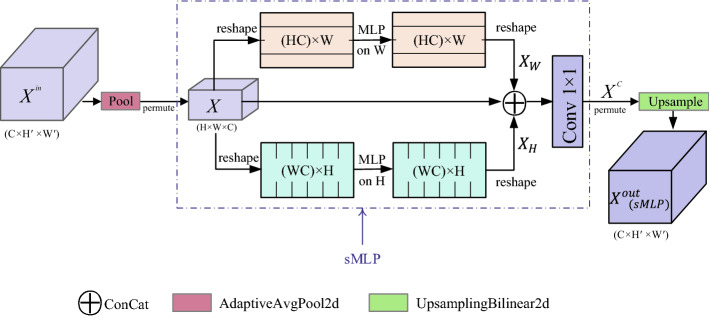
Overview of the sMLP block. (Created by ‘Microsoft Office Visio 2013’ url: https://www.microsoft.com/zh-cn/microsoft-365/previous-versions/microsoft-vision-2013).

In this paper, we add a stage of downsampling to the front of the sMLP block and a stage of upsampling to the back end of the sMLP block. We integrate the sampling session with the sMLP block and apply it to the network. The purpose of it is to extract the deep global dependencies of the samples and obtain richer feature encoding information. We show the sMLP block in Fig. 7. As is presented, the input vector is first down-sampled and then passed through the sMLP block. The sMLP consists of 3 branches. The upper branch is responsible for mixing information along horizontal direction, and the lower branch is responsible for mixing information along vertical direction. The middle branch is identity mapping. The outputs of three branches are concatenated, then processed by point-by-point conv (Conv 1 × 1), and finally upsampled to obtain the final output.

Specifically, Let $X^{in}$ ∈ $R^{{C \times H^{\prime } \times W^{\prime } }}$ denote the collection of input tokens. Firstly, downsampling and permuting is performed to obtain $X \in R^{H \times W \times C}$. As shown in Eq. (14):

14 $$ X = permute\left[ {down\left( {X^{in} } \right)} \right] $$

where $down$ and $permute$ represent downsampling and transpose operations respectively. The Eq. (15), (16) and (17) show the size change.

15 $$ H^{\prime } \times W^{\prime } = 4 \times H \times W $$

16 $$ H^{\prime } = 2 \times H $$

17 $$ W^{\prime } = 2 \times W $$

where $H^{\prime }$ and $W^{\prime }$ represent the input size, and $H$ and $W$ represent the size after downsampling.

In sMLP's upper branch (horizontal mixing path), the data tensor is reshaped into (*HC*) × *W*, and a linear layer with weights $W_{W} \in R^{W \times W}$ is applied to each of the (*HC*) rows to mix information. Similar operation is applied in lower branch (vertical mixing path) and the linear layer is characterized by weights $W_{H} \in R^{H \times H}$. Finally, the outputs of three branch paths are fused together by cascading, processed by conv1 × 1. The output is $X^{C} \in R^{H \times W \times C}$. As follows Eq. (18):

18 $$ X^{C} = FC\left( {concat\left( {X_{H} ,X_{W} ,X} \right)} \right) $$

where $concat$ means to concatenate the outputs (i.e., $X_{H} ,X_{W} \;and \;X$) of the three branches by channel, and $FC$ stands for conv1 × 1.

Restore the $X^{C}$ dimension to $R^{C \times H \times W}$. Finally, as shown in Eq. (19), the overall upsampling doubles length and width dimensions, thereby restoring the output size to *Hʹ* × *Wʹ*. That is, the final output of sMLP block is $X^{out}_{{\left( {sMLP} \right)}} \in R^{{C \times H^{\prime } \times W^{\prime } }}$.

19 $$ X^{out}_{{\left( {sMLP} \right)}} = up\left( {permute\left( {X^{C} } \right)} \right) $$

With sMLP block, we aggregate information along the axial direction individually and implement global dependence modeling to obtain richer feature coding information. We take advantage of GPA and Transformer as co-encoder, then enhance the global information modeling by sMLP block. Therefore, our GPA-TUNet adequately co-encodes local information dependence and global relevance dependence to obtain excellent performance for medical image segmentation.

### Decoder of GPA-TUNet

Similar to TransUNet^13^ settings, we adopt upsampling, dilated convolutions and skip connections to restore the original resolution and obtain prediction images.

## Experiments and analysis

### Datasets

(1) Synapse multi-organ segmentation dataset. Synapse is a multi-organ segmentation dataset containing 30 abdominal clinical CT cases. Following^13^, the 30 cases were randomly divided by us into 18 training cases and 12 testing cases. Each image annotation contains eight organs (aorta, gallbladder, left kidney, right kidney, liver, pancreas, spleen and stomach). (2) Automated cardiac diagnosis challenge dataset. ACDC is a public cardiac MRI dataset of 100 cases. As in^13^, 70 training samples, 10 validation samples and 20 testing samples are randomly divided for experiments. Each exam contains two different modalities, with corresponding labels including left ventricle (LV), right ventricle (RV), and myocardium (MYO). We use Dice Similarity Coefficient (DSC(%)) and 95% Hausdorff Distance (HD95(mm)) to evaluate our method on the two datasets.

### Implementation details

All experiments were performed on NVIDIA Corporation GV100 [TITANV] GPU, and input resolution is set to 224 × 224 for all experiments, with data expansion including random flips and rotations.

In hybrid encoder, the ViT^11^ (denoted as "R50-ViT") is combined with ResNet-50^1^ and 12 Transformer layers. In our co-encoder, we combine ViT and the proposed GPA attention. The GPA was conducted using groups of 2 to divide the horizontal and vertical axes. In order to reduce the computational complexity and cost of the model, the sMLP block at the end of the encoder is performed in a single block. For cascading upsampling blocks, we are consistent with^13^, i.e., concatenation of four 2 × upsampling blocks in succession is performed to achieve full resolution. In this paper, the input resolution is set as 224 × 224 and patch size P is 16, except for special cases. And for the training of the model is using SGD optimizer with learning rate of 0.01, momentum of 0.9 and weight decay of 1e-4. For both Synapse and ACDC datasets, we have default batch size of 12. And default iterations are 20 k and 14 k respectively on the two datasets. All experiments were performed using a single NVIDIA Corporation GV100 [TITANV] GPU.

Same as^5,6^, we extrapolate all 3D volumes slice-by-slice and all predicted 2D slices are stacked to reconstruct 3D predictions for evaluation.

### Loss functions

In medical image segmentation, the area of background region is much larger than foreground region. If foreground information is misjudged as background information, acc score will be very high, but the actual segmentation effect is not proportional to acc score. Therefore, in the field of medical image segmentation, single loss function often cannot reflect the performance of the model. Following TransUNet^13^, our model invokes two loss functions: *CrossEntropyLoss* and *Dice Loss*. The Eq. (20) and (21) demonstrate the calculation of *CrossEntropyLoss* and *Dice Loss* respectively.

20 $$ {\mathcal{L}}_{CrossEntropyLoss} = - \mathop \sum \limits_{x} \left( {p\left( x \right)logq\left( x \right)} \right) $$

where $q\left( x \right)$ stands for ground-truth label, $p\left( x \right)$ stands for predictive value.

21 $$ {\mathcal{L}}_{DiceLoss} = \frac{2TP}{{FP + 2TP + FN}} $$

where $TP$ is true-positive, $FP$ is false-positive, $FN$ is false-negative.

The ratio of the *CrossEntropyLoss* and *Dice Loss* is $\lambda_{1} :\lambda_{2}$. The *total*-*loss* of the network is as follows Eq. (22).

22 $$ {\mathcal{L}}_{total - loss} = \lambda_{1} *{\mathcal{L}}_{CrossEntropyLoss} + \lambda_{2} *{\mathcal{L}}_{DiceLoss} $$

We set $\lambda_{1} = \lambda_{2} = 0.5$ in this paper. The influence of *CrossEntropyLoss* and *Dice Loss* ratio for model performance is discussed in our ablation study.

### Evaluation metrics

Consistent with baseline^13^, DSC(%) and HD95(mm) were used as evaluation metrics to evaluate performance of the model. DSC(%) is used to measure the similarity between Prediction and Ground Truth. HD95(mm) is defined as the quantized value of 95% of the maximum distance of the surface distance between Prediction and Ground Truth. The calculation methods of DSC(%) and HD95(mm) are shown in Eq. (23) and Eq. (24), respectively.

23 $$ DSC = \frac{{2 \times \left| {X \cap Y} \right|}}{\left| X \right| + \left| Y \right|} $$

24 $$ HD95 = max_{k95\% } \left[ {d\left( {X,Y} \right),d\left( {Y,X} \right)} \right] $$

where $X$ and $Y$ represent the Prediction and Group Truth, respectively.

### Experimental results

The experimental results on the Synapse and ACDC datasets are shown in Tables 1 and 2. In this paper, the highlighted part of tables indicates the best performance value, we will not specifically address this point in subsequent narrative.

**Table 1:** Experimental results of the Synapse Dataset.

Method	DSC(%)	HD95(mm)	Aorta	Gallbladder	Kidney(L)	Kidney(R)	Liver	Pancreas	Spleen	Stomach
V-Net^25^	68.81	–	75.34	51.87	77.10	**80.75**	87.84	40.05	80.56	56.98
DARR^41^	69.77	–	74.74	53.77	72.31	73.24	94.08	54.18	89.90	45.96
R50 UNet^2^	74.68	36.87	84.18	62.84	79.19	71.29	93.35	48.23	84.41	73.92
R50 AttnUNet^9^	75.57	36.97	55.92	63.91	79.20	72.71	93.56	49.37	87.19	74.95
UNet^2^	76.85	39.70	89.07	**69.72**	77.77	68.60	93.43	53.98	86.67	75.58
UNet + + ^7^	78.13	25.65	89.27	62.35	83.00	78.98	94.53	56.70	85.99	74.20
UNet3 + ^8^	73.81	30.82	86.32	59.06	79.16	71.26	93.13	46.56	84.94	70.08
Att-UNet^42^	77.77	36.02	**89.55**	68.88	77.98	71.11	93.57	58.04	87.30	75.75
R50 ViT^13^	71.29	32.87	73.73	55.13	75.80	72.20	91.51	45.99	81.99	73.95
ViT^11^	61.50	39.61	44.38	39.59	67.46	62.94	89.21	43.14	75.45	69.78
TransUNet^13^	77.48	31.69	87.23	63.13	81.87	77.02	94.08	55.86	85.08	75.62
SwinUNet^20^	79.13	21.55	85.47	66.53	83.28	79.61	94.29	56.58	**90.66**	76.60
MT-UNet^43^	78.59	26.59	87.92	64.99	81.47	77.29	93.06	59.46	87.75	76.81
GPA-TUNet (Ours)	**80.37**	**20.55**	88.74	65.63	**83.51**	80.37	**94.84**	**63.89**	87.58	**78.40**

DSC(%) of each single class is also presented.

Significant values are in [bold].

**Table 2:** Experimental results of the ACDC Dataset.

Methods	DSC(%)	RV	Myo	LV
R50 U-Net^2^	87.55	87.10	80.63	94.92
R50 Att-UNet^9^	86.75	87.58	79.20	93.47
CE-Net^44^	87.21	85.68	83.97	91.98
UNet^2^	88.28	86.08	86.04	92.72
UNet + + ^7^	89.06	87.66	86.47	93.06
UNet3 + ^8^	88.28	86.08	86.04	92.72
R50 ViT^13^	87.57	86.07	81.88	94.75
TransUNet^13^	89.71	88.86	84.53	95.73
SwinUNet^20^	90.00	88.55	85.62	**95.83**
UNETR^18^	88.61	85.29	86.52	94.02
GPA-TUNet (Ours)	**90.37**	**89.44**	**87.98**	93.68

Significant values are in [bold].

As is shown in Table 1, traditional CNN still has better performance, with Att-UNet even outperforming TransUNet. However, our method greatly outperforms CNN-based methods (UNet, etc.), attention mechanism-based methods (Att-UNet, etc.) and Transformer-based methods. On the Synapse dataset, mean DSC(%) and HD95(mm) of our method (GPA-TUNet) reached 80.37% and 20.55 respectively, it has obtained the optimal mean DSC(%) and HD95(mm). Compared with baseline (TransUNet), DSC(%) and HD95(mm) performance improved by 2.89% and 11.14% respectively. Our method obtained the current optimum on 4 organs (Kidney (L) etc.). As shown in Fig. 11, our model has significant performance in the segmentation of organs with large areas and organs with large axial spans. For example, the segmentation of pancreas in line 2 and stomach in line 3. The reason is that large organs have a large area, a large axial span and many reference pixels. GPA attention and sMLP are local and global models based on axial information. Therefore, for GPA and sMLP, there are more axial reference data for large organs, so the network has strong learning ability, important information is not easily lost, and has prominent axial perception ability. Combination of GPA and sMLP can better capture the local and global correlation of samples. As a result, GPA-TUNet has mighty capacity for segmentation of organs with a large area and a large axial span.

As is shown in Table 2, On the ACDC dataset, mean DSC(%) of our method (GPA-TUNet) reached 90.37%. Compared with baseline (TransUNet), DSC(%) performance improved by 0.66%. It has excellent segmentation performance on RV and Myo. From Fig. 12, GPA-TUNet shows weaker under-segmentation and over-segmentation. It has significant performance compared with other methods, which is consistent with the quantitative experimental results in Table 2.

**Figure 8:**
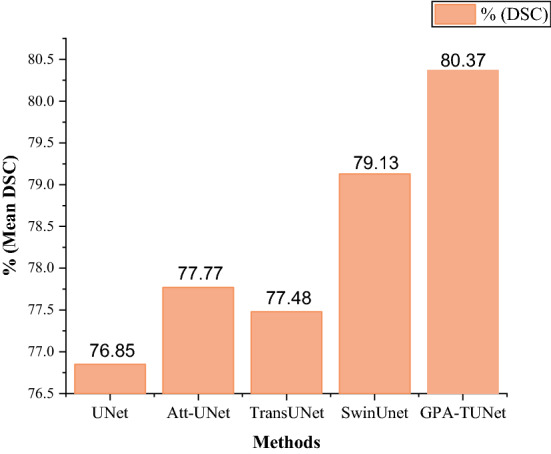
Comparison of mean DSC(%) based on different methods on the Synapse dataset. Segmentation performance comparison of classical architectures based on several different methods. (Created by ‘Origin and OriginPro 2021’ url: https://www.originlab.com/origin).

To further demonstrate the performance and advantages of GPA-TUNet, we compared the mean DSC(%) for several classical models (UNet, Att-UNet, TransUNet, SwinUNet) under different approaches on the Synapse dataset. The results are shown in Fig. 8. It is obvious from Fig. 8 that our GPA-TUNet has made outstanding progress compared with several classical networks, and also has the highest DSC(%) result.

### Analytical study

#### On the influence of GPA and sMLP block

To better evaluate the proposed GPA-TUNet framework and to verify the effectiveness of its new approach to GPA with sMLP block on performance, we compared our model with baseline (TransUNet^13^).

#### Synapse dataset

**Figure 9:**
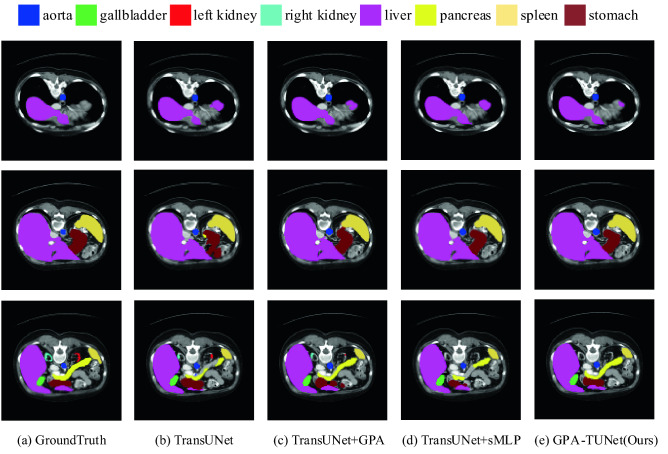
Ablation segmentation results of GPA and sMLP block on the Synapse dataset. From left to right: (**a**) Ground Truth, (**b**) TransUNet, (**c**) TransUNet + GPA, (**d**) TransUNet + sMLP, (**e**) GPA-TUNet(Ours). (Created by ‘Microsoft Office Visio 2013’ url: https://www.microsoft.com/zh-cn/microsoft-365/previous-versions/microsoft-vision-2013).

As can be seen from Table 3, neither GPA nor sMLP block is dispensable for the model, as removing either of them may result in performance loss. Applying all of them to the network, DSC(%) increased by 2.89%. We also provide the ablation qualitative results for the Synapse dataset, as shown in Fig. 9. It can be seen that both GPA and sMLP block play a certain role in improving network performance. When they are applied to network simultaneously, the segmentation result is improved more obviously. Therefore, the qualitative results of Fig. 9 agree with our quantitative results in Table 3. In addition, combined with Fig. 11, we found that GPA-TUNet had outstanding segmentation performance in large organs and organs with large axial span. The reasons are as follows. Big organs have large span in axial direction, while GPA is exactly axial attention, which carries out local modeling from different directions (horizontal and vertical). Therefore, GPA enhances the network's attention to the axial local foreground information, weakens other background information, and reduces misjudgment. Further, sMLP is global modeling based on axial direction. Therefore, the combination of GPA and sMLP greatly enhances the modeling ability of axial information, thus focuses on improving the segmentation performance of large organs and organs with large axial span. From Table 3, we further show that GPA and sMLP Block have the effect of promoting each other's network performance, and the segmentation accuracy is not just a simple superposition of the two. The same condition applies to HD95(mm) evaluation metric. HD95(mm) decreases by 7.39% and 2.16% compared to TransUNet when GPA and sMLP block are applied to the network alone. Applying all of them to the network, HD95(mm) significantly decreased by 11.14%.

**Table 3:** Ablation study on the Synapse dataset.

Method	DSC(%)	HD95(mm)
TransUNet^13^	77.48	31.69
TransUNet + GPA	78.88	24.30
TransUNet + sMLP	78.01	29.53
GPA-TUNet (Ours)	**80.37**	**20.55**

Significant values are in [bold].

#### ACDC dataset

**Figure 10:**
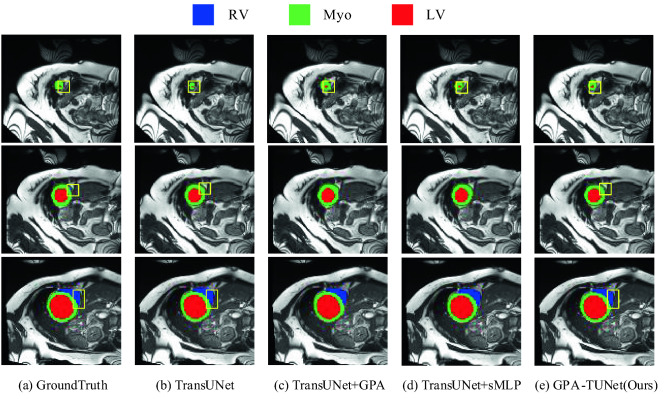
Ablation segmentation results of GPA and sMLP block on the ACDC dataset. From left to right: (**a**) Ground Truth, (**b**) TransUNet, (**c**) TransUNet + GPA, (**d**) TransUNet + sMLP, (**e**) GPA-TUNet(Ours). (Created by ‘Microsoft Office Visio 2013’ url: https://www.microsoft.com/zh-cn/microsoft-365/previous-versions/microsoft-vision-2013).

The ablation performance of GPA-TUNet on the ACDC dataset is shown in Table 4. We can see that, like the Synapse dataset, GPA and sMLP Block are not dispensable to the model, both of which improve the model segmentation performance. Applying all of them to the network, DSC(%) increased by 0.66%. We provide the ablation qualitative results for the ACDC dataset, as shown in Fig. 10. As can be seen from Fig. 10, when GPA and sMLP Block are jointly applied to network, the optimal segmentation result is achieved in our GPA-TUNet. It reduces more under-segmentation (such as the Myo on the first line, the RV on the second line) and over-segmentation (such as the RV on the third line).

**Table 4:** Ablation study on the ACDC dataset.

Method	DSC(%)	RV	Myo	LV
TransUNet^13^	89.71	88.86	84.53	**95.93**
TransUNet + GPA	89.82	88.25	87.73	93.49
TransUNet + sMLP	89.82	89.40	86.39	93.68
GPA-TUNet (Ours)	**90.37**	**89.44**	**87.98**	93.68

Significant values are in [bold].

For Figs. 9 and Fig. 10, we especially emphasize that in the comparison of TransUNet + GPA and TransUNet segmentation result graphs, we see that GPA plays an important role in highlighting local foreground information and weakening background information. For example, when the liver is segmented in the first row in Fig. 9, TransUNet + GPA shows a suppressing effect on the false positives of TransUNet, that is, GPA weakens the background information. For the segmentation of stomach in the second row and pancreas in the third row in Fig. 9, TransUNet + GPA promotes the partial information that is not segmented by TransUNet, that is, GPA highlights the local foreground information. A similar situation can also be seen in Fig. 10. For example in the first row for the segmentation of Myo, TransUNet + GPA shows a boost compared to TransUNet. Therefore, through the segmentation graphs of TransUNet + GPA and TransUNet on Synapse and ACDC datasets, we further verify the ability of GPA attention to highlight local foreground information and weaken background information as mentioned earlier.

#### On the influence of input resolution

The default input resolution for GPA-TUNet is 224 × 224. As shown in Table 5, we show the results of training GPA-TUNet at resolutions of 288 × 288 and 352 × 352. When input resolution is 288 × 288, patch size remains the same (i.e., 16), which results in an increase of approximately 1.65 × 1.65 in the length of the transformer sequence. Since shortly of the added sequence length compared to input size of 224 × 224, we find that the performance improvement is not significant. DSC(%) has only increased by 0.25%. However, when the resolution is changed from 224 × 224 to 352 × 352 will result in DSC(%) performance improvement of 1.00%. As pointed out by^11^, increasing the input resolution can lead to more significant performance improvements. Higher resolution means that we will trade a larger computational cost for an increase in average DSC(%). Due to GPU memory resource limitations, we no longer train GPA-TUNet results on 512 × 512 at high resolution. Therefore, based on the computational cost and memory limitation to consider, we determine the experiments at 224 × 224 resolution to demonstrate the validity and reliability of GPA-TUNet. We show the mean DSC(%) of the Synapse dataset for different input image resolutions in Table 5, which also further shows the segmentation accuracy about eight organs.

**Table 5:** Ablation study on the influence of input resolution of the Synapse Dataset.

Resolution	DSC (%)	HD95 (mm)	Aorta	Gallbladder	Kidney(L)	Kidney(R)	Liver	Pancreas	Spleen	Stomach
224	80.37	**20.55**	88.74	65.63	83.51	80.37	94.84	63.89	87.58	78.40
288	80.62	24.85	**89.20**	64.06	82.05	77.67	**95.49**	63.19	90.66	**82.63**
352	**81.37**	21.46	88.50	**67.98**	**84.67**	**82.44**	94.78	**65.01**	**90.80**	76.79

Significant values are in [bold].

#### On the influence of loss function (evaluation metrics)

In order to test the effectiveness of the two loss functions, we adjusted the *CrossEntropyLoss* and *Dice Loss* occupancy ratios and performed the corresponding ablation experiments. The experimental results are shown in Table 6.

**Table 6:** Ablation study on the influence of loss function ratio of the Synapse Dataset.

*CrossEntropyLoss* ratio ($\lambda_{1}$)	*DiceLoss* ratio ($\lambda_{2}$)	DSC (%)	HD95(mm)	Aorta	Gallbladder	Kidney(L)	Kidney(R)	Liver	Pancreas	Spleen	Stomach
0.2	0.8	79.83	37.54	87.56	65.24	79.93	74.89	94.70	63.47	**91.07**	**81.77**
0.3	0.7	79.73	29.69	87.84	58.87	83.30	78.67	94.59	**65.51**	89.59	79.49
0.4	0.6	80.18	26.93	88.36	62.17	84.17	80.07	94.73	62.03	90.57	79.36
**0.5**	**0.5**	**80.37**	**20.55**	**88.74**	**65.63**	83.51	80.37	**94.84**	63.89	87.58	78.40
0.6	0.4	80.01	27.01	87.18	64.26	83.08	79.30	94.61	62.68	90.18	78.79
0.7	0.3	80.04	28.05	87.05	62.47	83.33	80.37	94.52	63.20	90.46	78.93
0.8	0.2	79.86	28.44	86.69	64.54	**85.26**	**80.90**	94.84	61.94	88.03	76.66

Significant values are in [bold].

From Table 6, we can find that the proportion of the loss function has little effect on the experimental results. The segmentation achieved at a *CrossEntropyLoss* to *Dice Loss* ratio of 1:1 is optimal, with mean DSC(%) and HD95(mm) were 80.37% and 20.55 respectively. We found that increasing the proportion of *Dice Loss* to 0.6, 0.7 and 0.8 respectively brought a decrease of 0.19%, 0.64% and 0.54% to DSC(%); increasing the proportion of *CrossEntropyLoss* to 0.6, 0.7 and 0.8 respectively brought a decrease of 0.36%, 0.33% and 1.51% to DSC(%). As for HD95(mm), we can see from Table 6 that HD95(mm) gradually becomes larger as the ratio moves away from 1:1. That is, the performance gradually deteriorates. Therefore, maintaining the ratio of *CrossEntropyLoss* to *Dice Loss* at 1:1 seems to give the best results for our model. All other experiments were performed under this condition.

#### On the influence of attention mechanism

As shown in Tables 7 and 8, in order to explore the impact of different attention mechanisms on model performance, we list the experimental results of adding different attention mechanisms to baseline (TransUNet^13^) on the Synapse and ACDC datasets. SE stands for SE attention (from^45^), SK stands for SK attention (from^46^), CA stands for Coordinate Attention (from^47^), ECA stands for Efficient Channel Attention (from^48^), CBAM stands for Convolutional Block Attention Module attention (from^49^). As shown in Table 7, our TransUNet + GPA performs well on the Synapse dataset, DSC(%) reached the highest score, and HD95(mm) result is slightly lower than TransUNet + SK. As shown in Table 8, TransUNet + SE reached the state-of-the-art performance on the ACDC dataset, and our TransUNet + GPA is 0.27% lower than it. However, our TransUNet + GPA undoubtedly outperforms TransUNet, TransUNet + SK and TransUNet + ECA. Therefore, the combined results show that our GPA attention has an excellent positive effect on performance of our medical image segmentation model. GPA allows us to match or even exceed some current attentions in our medical image segmentation task.

**Table 7:** Ablation study on the influence of Attention Mechanism of the Synapse Dataset.

Method	DSC(%)	HD95(mm)	Aorta	Gallbladder	Kidney(L)	Kidney(R)	Liver	Pancreas	Spleen	Stomach
TransUNet^13^	77.48	31.69	87.23	63.13	**81.87**	77.02	94.08	55.86	85.08	75.62
TransUNet + SE	78.53	31.05	87.00	**66.02**	81.38	77.54	93.79	60.48	86.47	75.56
TransUNet + SK	77.51	**23.62**	86.66	61.90	78.64	77.26	**94.57**	55.73	87.33	**78.02**
TransUNet + CA	77.62	32.91	87.27	59.70	79.38	75.69	93.45	**61.29**	**88.31**	75.88
TransUNet + ECA	77.45	30.74	87.19	63.76	81.53	**80.06**	93.69	57.52	82.98	72.91
TransUNet + CBAM	78.41	32.04	87.51	63.89	81.34	77.00	93.90	59.21	88.22	76.21
TransUNet + GPA(Ours)	**78.88**	24.30	**87.79**	65.46	80.54	79.12	93.94	60.70	87.67	75.81

Significant values are in [bold].

**Table 8:** Ablation study on the influence of Attention Mechanism of the ACDC Dataset.

Methods	DSC(%)	RV	Myo	LV
TransUNet^13^	89.71	88.86	84.53	**95.73**
TransUNet + SE	**90.09**	**89.72**	86.97	93.59
TransUNet + SK	89.47	88.83	86.55	93.04
TransUNet + CA	90.06	89.69	87.17	93.32
TransUNet + ECA	89.64	88.81	86.69	93.41
TransUNet + CBAM	89.97	89.42	86.86	93.64
TransUNet + GPA(Ours)	89.82	88.25	**87.73**	93.49

Significant values are in [bold].

### Visualizations

**Figure 11:**
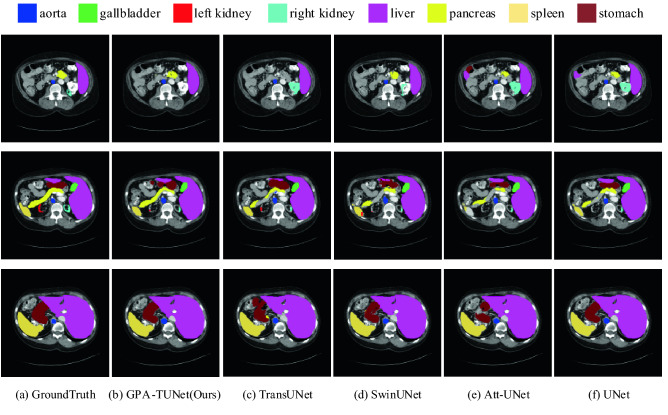
The segmentation results of different methods on the Synapse dataset. From left to right: (**a**) Ground Truth, (**b**) GPA-TUNet (Ours), (**c**) TransUNet, (**d**) SwinUNet, (**e**) Att-UNet, (**f**) UNet. (Created by ‘Microsoft Office Visio 2013’ url: https://www.microsoft.com/zh-cn/microsoft-365/previous-versions/microsoft-vision-2013).

**Figure 12:**
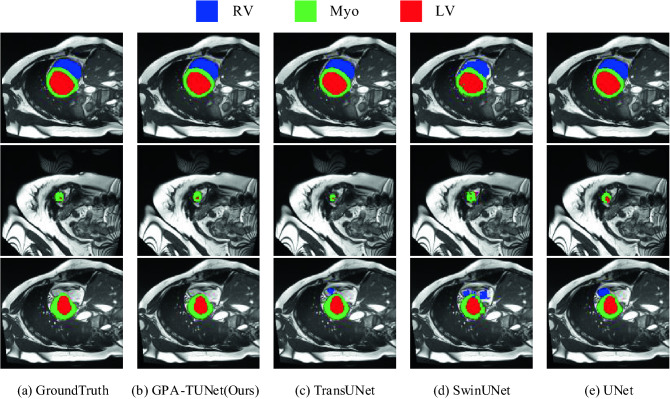
The segmentation results of different methods on the ACDC dataset. From left to right: (**a**) Ground Truth, (**b**) GPA-TUNet (Ours), (**c**) TransUNet, (**d**) SwinUNet, (**e**) UNet. (Created by ‘Microsoft Office Visio 2013’ url: https://www.microsoft.com/zh-cn/microsoft-365/previous-versions/microsoft-vision-2013).

Qualitative comparison results for Synapse and ACDC datasets are provided to visualize the segmentation performance of GPA-TUNet, as shown in Figs. 11 and 12.

#### Synapse dataset

From Fig. 11, it can be seen that: 1) UNet and Att-UNet based on pure CNN methods are more likely to result in over-segmentation (e.g., segmentation of right kidney and liver in the first row) or under-segmentation (e.g., segmentation of pancreas and spleen in the second row) of the organs. 2) These conditions are improved in TransUNet with the addition of Transformer. This suggests that the hybrid Transformer-based model has a stronger ability to encode global context and make semantic distinctions. However, it can be seen from Fig. 11 that the overall segmentation of the SwinUNet network based on the pure Transformer is not satisfactory. 3) Compared with other methods, GPA-TUNet has a better segmentation effect. For example, the split between right kidney and liver in the first row did not show a false positive. The segmentation of pancreas in the second row and stomach in the third row gives significantly better results than other methods.

#### ACDC dataset

The qualitative experiment of GPA-TUNet on the ACDC dataset is shown in Fig. 12. It is obvious from Fig. 12 that the segmentation effect of GPA-TUNet is closer to Ground Truth. For example, GPA-TUNet is obviously superior to other methods for segmentation of Myo and LV in the second row. It showed no false positives for segmentation of RV in the third line.

In addition, we found that GPA-TUNet showed exceptional ability in segmenting big organs and organs with large width or height spans on two datasets. The reasons are as follows. Large organs have large axial span, for GPA attention, there are more axial reference pixels in Eqs. (2) and (4), so that the axial prospect information can be more accurately grasped and local modeling can be carried out. And sMLP is global modeling based on feature maps. GPA is combined with sMLP, axial local modeling and global modeling are incorporated into learning process, which enhances the learning ability of the network. Therefore, compared with baseline (TransUNet), we performed superior for organ segmentation with a larger axial span. It benefits from the joint modeling of GPA and Transformer, as well as the global modeling of sMLP. The above quantitative experimental results (Tables 1 and 2) also verify the effectiveness of GPA attention and sMLP for the network as described previously.

These observations show that GPA-TUNet, jointly encoded by GPA Attention and Transformer, is able to preserve local foreground details well while achieving global relevance modeling for more accurate segmentation. That is, GPA-TUNet implements joint modeling of local information dependence and global relevance dependence. It allows the model to enjoy the benefits of both low-level detail and high-level global contextual information, and also has the advantage of highlighting foreground information and weakening background information.

#### Generalization to other datasets

To demonstrate the model generalization capability of GPA-TUNet, we evaluated the MR dataset ACDC which aims to accomplish automatic heart segmentation. The results of the evaluation are shown in Table 2. We can see that our GPA-TUNet has been consistently improved compared to the CNN-based approach (UNet), the pure Transformer-based approach (SwinUNet) and the hybrid encoder-based approach (TransUNet). GPA-TUNet also has the highest DSC(%) on the ACDC dataset compared to various previous state-of-the-art methods. We also provide the qualitative comparison results for the ACDC dataset, as shown in Fig. 12. GPA-TUNet has the best performance compared with other methods on the ACDC MR dataset. This is consistent with our previous results in the Synapse CT dataset.

## Conclusions

In this paper, we propose a new attention mechanism: Group Parallel Axial Attention (GPA), which enables local information modeling by computing feature weights in parallel with attention in different directions (horizontal and vertical) of the image. Combining GPA with Transformer, co-encoder GPA-TUNet is constructed to explore performance for medical image segmentation. It not only obtains sample local correlations by GPA based on pixel level, but also encodes powerful global context by Transformer with image features as sequences. Furthermore, we introduce sMLP to strengthen the global information dependence, which relies on sparse connections and weight sharing. The whole structure adopts U-shape structure, thus also preserving the coarse-grained features of CNN. Extensive experiments demonstrate that GPA-TUNet jointly models local information dependencies and global correlation dependencies properly. It is especially remarkable for the segmentation performance of organs with large axial spans. Compared with various other existing methods, GPA-TUNet achieves optimal segmentation results on two different publicly available datasets. GPA-TUNet has poor segmentation performance for small organs. The reason is that there are fewer axial reference pixels for small organs, and the model easily misjudges foreground information as background information. For the shortcomings of this paper, we will continue to improve in the future.

## Data Availability

The Synapse and ACDC datasets are openly available at: https://www.synapse.org/#!Synapse:syn3193805/wiki/217789(accessed on 28 April 2022) and https://www.creatis.insa-lyon.fr/Challenge/acdc/(accessed on 28 April 2022).
